# Nearly 400 million people are at higher risk of schistosomiasis because dams block the migration of snail-eating river prawns

**DOI:** 10.1098/rstb.2016.0127

**Published:** 2017-04-24

**Authors:** Susanne H. Sokolow, Isabel J. Jones, Merlijn Jocque, Diana La, Olivia Cords, Anika Knight, Andrea Lund, Chelsea L. Wood, Kevin D. Lafferty, Christopher M. Hoover, Phillip A. Collender, Justin V. Remais, David Lopez-Carr, Jonathan Fisk, Armand M. Kuris, Giulio A. De Leo

**Affiliations:** 1 Department of Biology, Hopkins Marine Station, Stanford University, Pacific Grove, CA 93950, USA; 2 Marine Science Institute, and Department of Ecology, Evolution and Marine Biology, University of California, Santa Barbara, CA 93106, USA; 3 Department of Biology, Medaille College, Buffalo, NY 14214, USA; 4 Department of Veterinary Technology, Medaille College, Buffalo, NY 14214, USA; 5 Emmett Interdisciplinary Program in Environmental Resources, School of Earth, Energy, and Environmental Sciences, Stanford University, Stanford, CA 94305, USA; 6 Department of Ecology and Evolutionary Biology and Michigan Society of Fellows, University of Michigan, Ann Arbor, MI 48109, USA; 7 School of Aquatic and Fishery Science, University of Washington, Seattle, WA 98195, USA; 8 Western Ecological Research Center, U.S. Geological Survey, Santa Barbara, CA 93106, USA; 9 Division of Environmental Health Sciences, School of Public Health, University of California Berkeley, Berkeley, CA 94720, USA; 10 Department of Geography, University of California Santa Barbara, Santa Barbara, CA 93106, USA

**Keywords:** dam, disease control, schistosome, bilharzia, biological control, planetary health

## Abstract

Dams have long been associated with elevated burdens of human schistosomiasis, but how dams increase disease is not always clear, in part because dams have many ecological and socio-economic effects. A recent hypothesis argues that dams block reproduction of the migratory river prawns that eat the snail hosts of schistosomiasis. In the Senegal River Basin, there is evidence that prawn populations declined and schistosomiasis increased after completion of the Diama Dam. Restoring prawns to a water-access site upstream of the dam reduced snail density and reinfection rates in people. However, whether a similar cascade of effects (from dams to prawns to snails to human schistosomiasis) occurs elsewhere is unknown. Here, we examine large dams worldwide and identify where their catchments intersect with endemic schistosomiasis and the historical habitat ranges of large, migratory *Macrobrachium* spp. prawns. River prawn habitats are widespread, and we estimate that 277–385 million people live within schistosomiasis-endemic regions where river prawns are or were present (out of the 800 million people who are at risk of schistosomiasis). Using a published repository of schistosomiasis studies in sub-Saharan Africa, we compared infection before and after the construction of 14 large dams for people living in: (i) upstream catchments within historical habitats of native prawns, (ii) comparable undammed watersheds, and (iii) dammed catchments beyond the historical reach of migratory prawns. Damming was followed by greater increases in schistosomiasis within prawn habitats than outside prawn habitats. We estimate that one third to one half of the global population-at-risk of schistosomiasis could benefit from restoration of native prawns. Because dams block prawn migrations, our results suggest that prawn extirpation contributes to the sharp increase of schistosomiasis after damming, and points to prawn restoration as an ecological solution for reducing human disease.

This article is part of the themed issue ‘Conservation, biodiversity and infectious disease: scientific evidence and policy implications’.

## 1. Introduction

Dams stabilize water availability, generate power and facilitate navigation, and thus provide substantial benefits to human society, but they sometimes lead to disease epidemics [1]. One of the most famous dam-associated epidemics occurred in 1986 after the Diama Dam was installed on the Senegal River. The dam was intended to support agricultural expansion by stabilizing water availability and preventing saltwater intrusion. Within a few years, persistent and intense infections of human schistosomiasis (bilharziasis) were observed in upstream villages. The Diama Dam has been well studied, and compelling evidence suggests that increased transmission arose as the result of interacting ecological, epidemiological and socio-economic factors [2–4]. One hypothesis that has received less attention argues that the Diama Dam excludes native, migratory snail predators, notably *Macrobrachium* spp. river prawns, by blocking the predators' migrations. This causes snail populations to be released from a primary regulating force: predation pressure [5–7]. In a test of this hypothesis, a demonstration intervention in Senegal showed that restoring native *M. vollenhovenii* prawns to one village water-access point led to significantly lower reinfection rates for human schistosomiasis, compared to a nearby control village [5].

The association between dams and human schistosomiasis has been recognized since the early 20th century, even while the mechanisms behind the association remain unclear. The completion of the Aswan Low Dam in Upper Egypt drove irrigation expansion in the 1930s. Schistosomiasis in this region increased rapidly, rising in some areas from below 20% prevalence to 75% prevalence [8]. Though the link between dam construction and schistosomiasis was disputed at the time by engineers, additional outbreaks following irrigation expansion eventually led to scientific consensus on the issue, with the Executive Board of the World Health Organization in its fifth session (1950) declaring that ‘the danger to health entailed by the establishment of irrigation schemes in areas where bilharziasis is present’ should be made clear to governments and technical advice provided [8].

Since Aswan, the effects of dams on schistosomiasis transmission have been observed all over Africa. A systematic review and meta-analysis by Steinmann *et al*. [9] found increased schistosomiasis risk across sub-Saharan Africa for people living within 5 km of dam reservoirs, compared to those outside this zone (with risk ratios of 2.4 and 2.6 for *S. haematobium* and *S. mansoni*, respectively). Similarly, schistosomiasis risk was elevated for people living near irrigated lands, often supported by dams, compared with people living far from irrigated lands (with risk ratios of 1.1 and 4.7 for *S. haematobium* and *S. mansoni* respectively) [9].

The most common human schistosomes are *S. haematobium* and *S. mansoni*, which co-occur in sub-Saharan Africa, where an estimated 97% of schistosome-infected humans are located [9]. *Schistosoma* spp. parasites cause considerable morbidity where they are endemic, especially among children who tend to bear the heaviest parasite loads, and in young adults who can be chronically infected from childhood [10]. The disease, schistosomiasis, is characterized by chronic pain, stunted growth, anaemia, inflammation, cognitive impairments and increased HIV risk in women. Further serious consequences include infertility, elevated risk of bladder cancer and liver failure [11]. Schistosomiasis is a primary or contributory cause of death in approximately 10% of those who are chronically infected [10].

The schistosome life cycle begins with adult schistosome worms, which inhabit the blood vessels of their human hosts for many years. Eggs are shed in urine and faeces and, where sanitation is inadequate, these eggs can wash into natural surface waters, where they hatch and seek their obligate snail hosts, *Biomphalaria* spp. for *S. mansoni* and *Bulinus* spp. for *S. haematobium* [10]. In snails, the parasite larvae reproduce asexually, generating hundreds to thousands of cercariae per day, which are the free-swimming infectious propagules that seek vertebrate hosts and penetrate their skin. People are exposed to cercariae when they wash, bathe, fish, wade, swim, or otherwise contact fresh water where infected snails are present. Schistosomiasis risk can increase when water resource development causes environmental changes that favour the schistosome life cycle. Dams may facilitate schistosomiasis transmission by increasing the density of intermediate snail hosts through lower salinity, more stable water flows (which reduces shear forces harmful to schistosome larvae), increases in vegetation on which snails depend for food and habitat, and—as we suggest—reductions in predation on snails.

There are trade-offs between the health risks and the socioeconomic benefits of dams and other anthropogenic landscape changes, but to date, solutions to reduce the health risks associated with environmental change or degradation remain elusive [12]. Just 3 years after the WHO issued its original warning on schistosomiasis risks associated with irrigation schemes, the WHO Expert Committee on Bilharziasis observed: ‘In spite of a formal cautionary notice…it is obvious that co-operation between health administrations and the authorities responsible for irrigation has not…been achieved’ [8]. This failure to prevent or mitigate the health risks of water resources development has continued to the present day [13]. In part, this may be due to a lack of historical emphasis on the ecological mechanisms underlying dam-driven schistosomiasis outbreaks, which precludes well-informed solutions.

To assess whether prawn restoration might have widespread potential for schistosomiasis control, we ask whether the dam-associated prawn declines coincident with schistosomiasis increases observed in the Diama Dam watershed in Senegal might be generalizable across a broad geographical area. To address this hypothesis, we analysed freely available historical and geographical data on human schistosomiasis infections across sub-Saharan Africa (where the vast majority of schistosome infections occur today), coupled with spatial maps of river prawn habitats and dams on the continent. We followed with a global-scale analysis asking: if there were an association between dam-driven exclusion of migratory prawns and schistosomiasis, what proportion of the total human population-at-risk of schistosomiasis could theoretically benefit from restoring native prawns?

*Macrobrachium* spp. prawns have been shown to eat schistosome-hosting snail species and also to experience population declines after dam construction [4,5,7,14]. Migratory species of *Macrobrachium* prawns are strongly affected by dams that block their reproductive behaviour by preventing the downstream migrations of females and the upstream migrations of juveniles required to complete their catadromous life cycles. This leads to substantial reductions or local extirpation of prawn populations in upstream catchments [15]. While migratory prawns are not the only predators of snails in these ecosystems (other predators include fish, birds and many other vertebrates), migratory prawns are likely to be among the most heavily impacted by dams [15]. Although few data are available on prawn distributions or population abundances, anecdotal reports suggest *Macrobrachium* spp. prawns have declined upstream of the Diama Dam in Senegal [4], the Kossou Dam in Cote D'Ivoire [16], the Akosombo Dam in Ghana [17,18] and the Tucuri Dam in Brazil [19], among others. Declines in river prawns have also been reported due to overharvesting and pollution across their range, particularly for *M. rosenbergii* in Bangladesh, India, Indonesia, Malaysia, the Philippines and Thailand [20]. If prawn declines help explain schistosomiasis spikes following dam projects, then prawn restoration could help combat disease.

## 2. Methods

We examined countries where prawns, dams and schistosomiasis co-occur, estimating the number of people living: (i) in areas subject to endemic schistosomiasis and (ii) within watersheds that are part of the native, historical ranges of 24 *Macrobrachium* spp. prawns identified by the Food and Agriculture Organization of the United Nations (FAO) as large-bodied and migratory [21].

To delineate schistosomiasis-endemic areas, we digitized a published global map depicting the endemic distributions of the six most common human schistosomes using ArcGIS version 10.1. Our maps were digitized from a combination of published sources [22–24]. Maps provided by these sources were adapted from the original published schistosomiasis distribution maps dating to 1984, based on a survey of the member states of the World Health Organization [24].

Next, we delineated prawn habitat boundaries for 24 large, marketable, migratory *Macrobrachium* spp. prawns, based on the FAO species catalogue of shrimps and prawns [21]. We limited our analysis to prawns that are viable as fisheries (and thereby likely to be noted in the FAO source) and those prawn species that must migrate downstream to brackish water estuaries to reproduce, and that might therefore be extirpated above dams that block migratory pathways.

In the FAO species catalogue of shrimps and prawns [21], countries and regions in which each species had been reported (and usually also targeted in a local fishery) were listed, and these descriptions were translated into digital maps in ArcGIS v. 10.1. Next, we estimated the habitat boundaries of the 24 prawn species across their ranges using watershed boundaries and literature-derived estimates of minimum and maximum migration distances from the coast for *Macrobrachium* spp. prawns. Literature descriptions of migration distances [25–30] suggested that a boundary of 400 km linear distance from the sea, upstream along the river network, was a reasonable minimum boundary distance that would encompass a habitat area in which migratory *Macrobrachium* spp. prawns were ‘likely’ to be found in abundance if their migration paths were unhindered, i.e. ‘likely’ habitat. We further estimated that 1000 km was the maximum linear distance along river network paths that prawns could penetrate during their lifetimes, i.e. ‘possible’ habitat. This is conservative given that *Macrobrachium ohione* occurs well above that distance in the Mississippi River drainage [25,31,32]. Watershed networks were built using the Global Hydro1 K dataset, freely available from the United States Geological Survey [33]. Watershed topography often limited the prawn range boundaries with a natural barrier (such as a ridge or a large waterfall) well before the migration minimum or maximum of 400 km or 1000 km was reached.

We compared our estimated habitat ranges to georeferenced sightings of our 24 target *Macrobrachium* species, available from the Global Biodiversity Information Facility [34]. The sightings were not used in generating the ranges, and thus provided an independent dataset suitable for assessing the plausibility of our predictions. We overlaid the point sightings onto our estimated habitat ranges (electronic supplementary material, figure S1) and tested the association between *Macrobrachium* sightings and the proposed ranges using quadrat counting in the R package ‘spatstat’ [35,36]. As a supplemental exercise, we also tested for associations between the spatial intensity (*λ*) of *Macrobrachium* sightings and the proposed ranges as an inhomogeneous Poisson process with habitat ranges as a categorical covariate with three levels (within 400 km, between 400 km and 1000 km, and further than 1000 km) using the function ‘*ppm*’ (see electronic supplementary material, figure S1).

Additionally, we determined that *M. jelskii*, a South American species with abbreviated larval development, is capable of breeding in freshwater [37]. It is likely that some *M. jelskii* sightings beyond our predicted ranges were non-migratory, freshwater adapted populations, and therefore we conducted analyses both including and excluding *M. jelskii* sightings (see electronic supplementary material, figure S1).

After determining the plausibility of estimated prawn habitat ranges, we added to our maps the global distribution of large dams, acquired from the Global Reservoir and Dam (GRanD) Database, available from the Socioeconomic Data and Applications Center [38]. Dam catchments were visualized and delineated using the Network Analyst tool in ArcGIS, which allowed us to outline the boundaries of the watershed networks upstream of each dam.

In order to estimate the number of schistosomiasis cases preventable by prawn restoration, we used published Gridded Population of the World estimates for 2015, Version 3 (GPWv3) available from Nasa's SEDAC data portal [39] to calculate the current human population living within areas around the globe that are both endemic for schistosomiasis and occur within native prawn ranges. Using the Spatial Analyst extension in ArcGIS, we summed individual counts in the gridded population of the world coverage that intersected with polygons delineating schistosomiasis-endemic areas and prawn habitat ranges. We used the most conservative (400 km) and the most liberal (1000 km) prawn range assumptions (described above) to estimate a minimum and maximum number of people that might potentially benefit from prawn restoration.

For the statistical analysis, we calculated odds ratios (ORs) of schistosomiasis disease after:before dams, using the number of people infected out of the total number examined, reported in the freely available Global Neglected Tropical Diseases (GNTD) Database [40]. Data were grouped within each of three scenarios: (i) people living within prawn habitat and upstream of large dams, (ii) people living within prawn habitat but in nearby undammed watersheds, or (iii) people living outside prawn habitat but within dammed catchment areas. We hypothesized that dams within prawn ranges might experience the greatest increases in schistosomiasis after dam completion, because migratory snail-predator (prawn) exclusion by dams would increase snail densities and therefore intensify transmission from snails to humans. Ecological surveys to provide quantitative information on prawn abundance at these locations were not available, and therefore our statistical tests did not estimate a prawn effect directly. Instead, we measured schistosomiasis changes within and outside prawn habitat to test, indirectly, whether a decline in migratory aquatic snail predators after dam building was a plausible driver in the causal web that leads to a dam–schistosomiasis association. To control for other dam effects unrelated to a loss of migratory predators—for example, increases in aquatic vegetation, inundation, agricultural inputs, worker migrations and water contact—we included as many catchments as possible outside prawn habitat that had sufficient data before and after the dam was built. Undammed ‘control’ watersheds, nearest the dam catchments included in our study, were included to control for background changes in schistosomiasis in each region unrelated to the ecological changes associated with dam building, such as implementation of national control programmes, regional demographic, development, or behavioural changes.

Individual studies quantifying schistosomiasis prevalence (proportion of the number of people examined that were found positive) were selected based on their spatial locations (i.e. latitude and longitude of each record in the GNTD Database [40]). Studies were categorized as those conducted before each focal dam was constructed and those conducted afterwards, based on the year or years in which human subjects were tested for schistosomiasis. For undammed watersheds, studies were categorized as those conducted before versus after the closure date of the matched nearby dam within the same prawn habitat region. For example, in the Senegal River Basin, the Diama Dam was closed in 1986; therefore, all studies upstream of this dam were categorized as those conducted before versus after 1986, and all undammed watersheds in Senegal outside the dammed catchment were included as undammed controls, also categorized as those conducted before versus after 1986.

To test for an overall difference among the odds of schistosome infection for people in the three scenarios, we ran a generalized linear mixed model with binomial errors and a ‘logit’ link function (the glmer function in the lme4 package, R v. 3.1.2 [41]) to explore the interaction between time (before/after) and scenario (i.e. people living within prawn habitat and upstream of large dams, people living within prawn habitat but in nearby un-dammed watersheds, or people living outside prawn habitat but within dammed catchment areas). As in before–after–control–impact analyses, the interaction term in our generalized linear mixed model tests for differences in the rise or fall of schistosomiasis in response to dam-building among the three ‘treatments’, or scenarios. We included random effects of region to control for unmeasured differences among groups, attributable to unmeasured variables specific to each region (e.g. regions such as Senegal River Basin, Volta River Basin, or regions made up of dammed and undammed watersheds in nearby, contiguous countries such as Cameroon–Nigeria–Congo) and between the two most common human schistosome species, *S. haematobium* and *S. mansoni*. Upon finding a significant interaction (indicating significant differences in the change in schistosomiasis prevalence among scenarios), we calculated and reported overall odds ratios, after:before dams, within each comparison type, using a hierarchical model stratified between *S. haematobium* and *S. mansoni*. For this, we used a generalized linear mixed model with binomial errors and ‘logit’ link function (the glmer function in the lme4 package, R v. 3.1.2), with a single random effect of region. For visualization of variability among each catchment across regions and schistosome species, we calculated and reported odds ratios, after:before dams, and their confidence intervals stratified across each dam catchment (or undammed watershed), country, and schistosome species, using logistic regression. Odds ratios are a standard epidemiological measure of association between an exposure (e.g. living upstream of a dam within range of prawns) and an outcome (having schistosomiasis), often calculated and reported as part of logistic regression analyses and their mixed-model counterparts. Odds ratios are equal to unity (and/or their confidence intervals include unity) when there is no association between the exposure and outcome; otherwise, odds ratios more than unity indicate an association, and those less than unity a protective effect, between the exposure and outcome.

For two river basins—the Senegal River Basin and Volta River Basin—the basins were large enough and schistosomiasis data were sufficient to characterize schistosomiasis prevalence before and after construction of each dam across a long gradient (>1000 km) of river distances from the coast, from downstream of the dam to upstream reaches beyond the historical reach of prawns. The Senegal River Basin runs east to west, and the Volta River Basin runs north to south; we thus compared prevalence changes across bands of *ca* 2° longitude (Senegal River) and *ca* 2° latitude (Volta River), within each basin, respectively.

## 3. Results

Fourteen dammed catchments, covering more than 4 million km^2^ in 16 countries, were included in our analysis, and undammed watershed areas spanning 10 countries were included as controls (table 1). Schistosomiasis data pertinent to our three scenarios and available from the GNTD Database included 679 054 individual human subjects tested for *S. haematobium* and 462 859 for *S. mansoni* in 4976 and 3725 unique locations, respectively, within the African continent.

**Table 1.** Large dams/catchments of Africa occurring within historical prawn habitat, and comparable areas (undammed watersheds and dams outside prawn ranges) with sufficient available data on human schistosomiasis before and after dams to be included in this study. *Sh. = Schistosoma haematobium; Sm. = S. mansoni; Si. = S. intercalatum; Sg. = S. guineensis.*

region	countries of Africa assessed	*Schistosoma* spp.	large dam/catchment (river) in prawn range	dam size (km^2^ catchment)	year dam closed	comparable undammed watersheds; country(ies) where located	large dam/catchment (river) outside prawn range	year dam closed	dam size (km^2^ catchment)
E & S Africa	Botswana^b^	*Sh. Sm.*	—		—	—	—	—	—
E & S Africa	Kenya^a,b^	*Sh. Sm.*	Kiambere (Tana)	11 950	1987	Kenya/Somalia	—	—	—
			Masinga (Tana)	7247	1980	—	—	—	—
E & S Africa	Madagascar^a,b^	*Sh. Sm.*	—	—	—	—	—	—	—
E & S Africa	Mozambique^a,b^	*Sh. Sm.*	Chipembe (Montepuez)	365	1985	Mozambique	—	—	—
E & S Africa	Malawi^a^	*Sh. Sm.*	—	—	—	—	—	—	—
E & S Africa	Namibia^b^	*Sh. Sm.*	—	—	—	—	—	—	—
E & S Africa	South Africa^a,b^	*Sh. Sm.*	—	—	—	—	—	—	—
E & S Africa	Sudan	*Sh. Sm.*	—	—	—	—	Jebel-Aulia (White Nile)	1937	1 667 623
E & S Africa	Tanzania^a,b^	*Sh. Sm.*	Mtera (Pangani)	69 840	1966	Tanzania	—	—	—
E & S Africa	Uganda	*Sh. Sm.*	—	—	—	—	Jebel-Aulia basin (White Nile)	1937	1 667 623
E & S Africa	Zambia	*Sh. Sm.*	—	—	—	—	Cahora Bossa (Zambezi)	1974	1 068 215
			—	—	—	—	Kafue-Gorge (Kafue)	1973	153 294
			—	—	—	—	Itezhi-Tezhi (Kafue)	1977	106 966
E & S Africa	Zimbabwe^a,b^	*Sh. Sm.*	—	—	—	—	—	—	—
W Africa	Angola^a,b^	*Sh. Sm.*	—	—	—	—	—	—	—
W Africa	Benin^a,b^	*Sh. Sm.*	—	—	—	—	—	—	—
W Africa	Burkina Faso^a,b^	*Sh. Sm.*	Akosombo basin (Volta)	404 375	1965	—	Akosombo basin (Volta)	1965	404 375
W Africa	Cameroon^a,b^	*Sh. Sm. Si.*	M'bakaou (Djerem)	20 401	1971	Congo	—	—	—
W Africa	Cote D'Ivoire^a,b^	*Sh. Sm.*	Taabo (Bandama)	59 729	1979	Liberia/Cote D'Ivoire	—	—	—
W Africa	Congo^a^	*Sh. Sm.*	—	—	—		—	—	—
W Africa	Gabon^a,b^	*Sh. Sm. Sg.*	—	—	—		—	—	—
W Africa	Ghana^a,b^	*Sh. Sm.*	Akosombo (Volta)	404 375	1965	Benin/Togo	—	—	—
W Africa	Guinea^a,b^	*Sh. Sm.*	—	—	—	—	—	—	—
W Africa	Liberia^a,b^	*Sh. Sm.*	—	—	—	—	—	—	—
W Africa	Mali^a,b^	*Sh. Sm.*	Diama basin (Senegal)	440 416	1986	—	Diama basin (Senegal)	1986	440 416
W Africa	Mauritania^a,b^	*Sh. Sm.*	Diama (Senegal)	440 416	1986	—	Diama basin (Senegal)	1986	440 416
W Africa	Nigeria^a,b^	*Sh. Sm. Si.*	Oyan (Oyan)	9166	1983	Congo	—	—	—
W Africa	Senegal^a,b^	*Sh. Sm.*	Diama (Senegal)	440 416	1986	Senegal	Diama basin (Senegal)	1986	440 416
W Africa	Sierra Leone^a,b^	*Sh. Sm.*	—	—	—	—	—	—	—
W Africa	Togo^a,b^	*Sh. Sm.*	Nangbeto (Mono)	15 702	1987	Benin/Togo	—	—	—

^a^Schistosomiasis endemic zone overlaps with prawn range.

^b^One or more large dams overlap with prawn range.

Our mapped global distributions of native prawn habitat (figure 3*a*; electronic supplemental material, figures S1 and S2) were validated as plausible by comparison with an independent dataset of prawn sightings. Most of the reported prawn sightings from the GBIF dataset fell within our proposed prawn ranges, with 91% of sightings reported within the 1000 km range and 81% within the 400 km range. Furthermore, sightings were strongly associated with the predicted prawn habitat ranges (*p* < 0.001) based on quadrat-counting that compares the expected to observed density of sightings in each range. When *M. jelskii* was excluded from analyses, the evidence for association between sightings and ranges remained robust (*p* < 0.001). The estimated intensity of sightings (spatial intensity) also strongly supported the plausibility of our proposed prawn habitat ranges (electronic supplementary material, figure S1).

**Figure 1.**
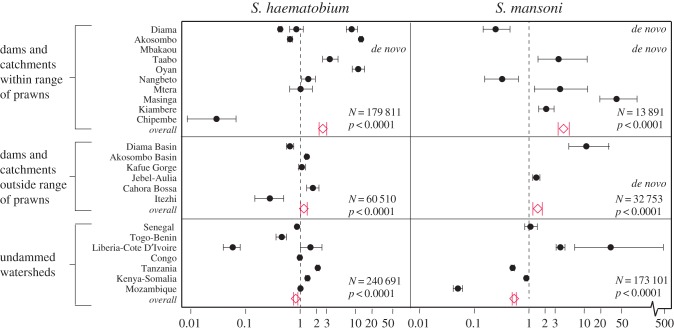
Odds ratios *after:before* dams within prawn ranges and comparison areas. Black circles show country-specific odds ratios and their confidence intervals among the respective dams and watersheds listed on the left. Red diamonds show the overall odds ratios and confidence intervals resulting from a generalized linear mixed model (with binomial errors) comparing *after* against *before* dam building for all dams/watersheds in each respective catchment/watershed category. *N* shows the total sample size (number of human subjects) for that category and the *p* value indicates the statistical significance of the test of the hypothesis that the overall odds ratios (diamonds) differ from unity. The vertical dashed line marks 1 : 1 odds ratios. Those points to the right of the dashed line indicate a significant increase in schistosomiasis after dam building; those to the left indicate a significant decline in schistosomiasis; and those overlapping the vertical dashed line show no change. *De novo* indicates a *de novo* introduction of the parasite in that catchment after the dam, whereby the presence of zero infections before the dam renders the odds ratio impossible to calculate. Note the log scale. (Online version in colour.)

Consistent with our hypothesis, the overall odds of schistosome infection increased within dammed catchments, with the highest increases overall among dams situated within native prawn habitat (figure 1, red diamonds). This effect was statistically supported by the fact that odds ratios for after:before dam building differed significantly among the three scenarios (*p* < 0.0001)—i.e. among (i) catchments within prawn habitat, (ii) undammed watersheds, and (iii) catchments outside prawn habitat. Those catchments located within prawn habitat had overall ORs after:before of 2.8 (2.7–2.9 CI) for *S. haematobium* and 4.4 (3.6–5.3 CI) for *S. mansoni*, (figure 1, red diamonds), demonstrating a strong overall increase in schistosomiasis. By contrast, undammed watersheds in nearby prawn habitat experienced overall schistosomiasis declines across the same time periods – ORs after:before 0.90 (0.88–0.91 CI) for *S. haematobium* and 0.75 (0.73–0.78 CI) for *S. mansoni* (figure 1, red diamonds). The dammed catchments outside prawn habitats also experienced schistosomiasis increases, but to a lesser degree than did dammed catchments within prawn ranges – ORs after:before 1.15 (1.1–1.2 CI) for *S. haematobium* and 1.5 (1.3–1.8 CI) for *S. mansoni* (figure 1, red diamonds).

**Figure 2.**
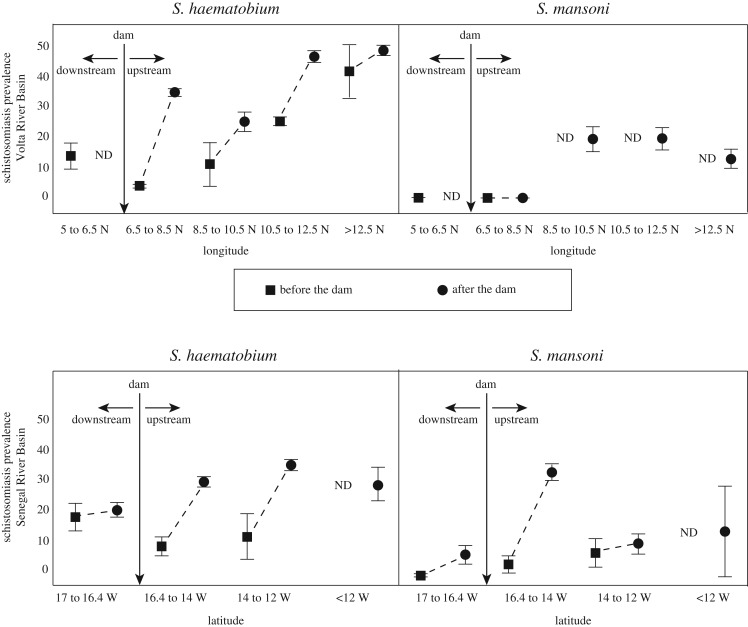
Schistosomiasis mean prevalences before (squares) and after (circles) the dams at (*a*) Akosombo (Volta River Basin, Ghana) and (*b*) Diama (Senegal River Basin, Senegal). *Schistosoma haematobium* and *S. mansoni* prevalences are stratified along latitudinal or longitudinal gradients that span the downstream, near upstream, and far upstream areas of the watersheds. Bars represent standard errors of the mean. ND = no data. Dashed lines connect the mean prevalences before and after the dam in each latitudinal/longitudinal band, to assist visualization of the temporal trend.

For the two dammed catchments where data were sufficiently resolved to explore how dam-related changes in human schistosomiasis differ from downstream to far-upstream habitats, plots of schistosomiasis prevalence before and after dam construction revealed that the greatest increases in prevalence occurred just upstream of the dams, and that increases became more moderate as distance upstream increased. This effect was actually mitigated by gradients in pre-dam prevalence, with lowest pre-dam prevalence found closer to the coast in both Senegal and Ghana (figure 2).

Schistosomiasis increases extended far inland, beyond the boundaries of the reservoirs above each dam. Specifically, for *S. haematobium* in the Volta River Basin, prevalence increases were evident from the Akosombo Dam at 6.5°N latitude all the way to the northern border with Burkina Faso, just above 11°N latitude (more than 550 km inland from the coast). By contrast, above 12°N latitude in the same catchment (where prawns were predicted to be historically rarer or absent in the basin), the *S. haematobium* prevalence in the Volta River Basin did not change before and after the construction of the Akosombo Dam. Similarly, in the Senegal River Basin, *S. mansoni* increased most dramatically just inland (east) of the Diama Dam at 16.4°W longitude, and was nearly unchanged below the dam and beyond 350 km inland, i.e. past 14°W latitude (figure 2). Mean *S. haematobium* prevalence, similarly, was static below the Diama Dam in Senegal, but increased dramatically upstream of the dam, from just above the dam to more than 600 km inland (east) to 12°W longitude (figure 2).

**Figure 3.**
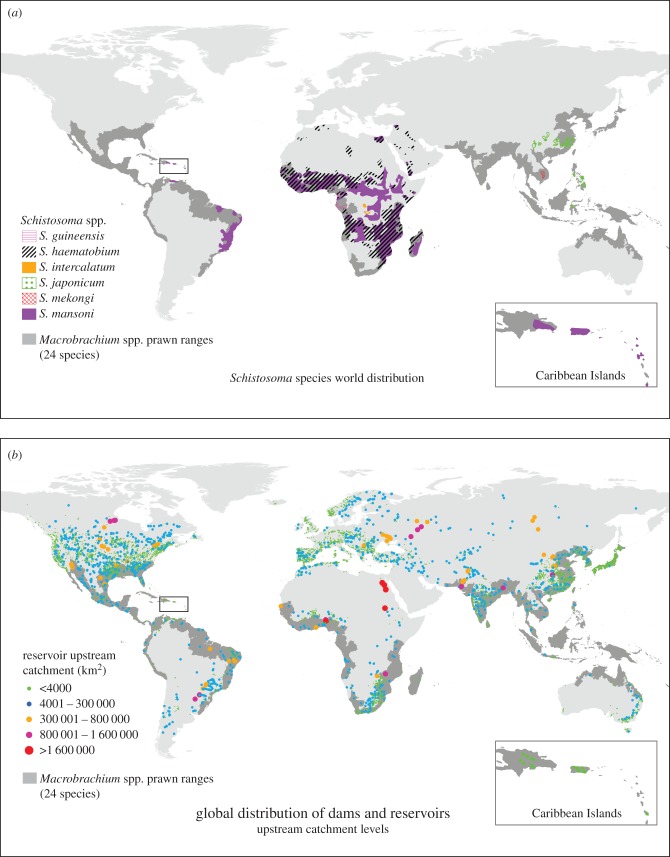
(*a*) *Schistosoma* spp. parasite world distributions (adapted from [22–24]) and *Macrobrachium* spp. prawn world distributions (data from this paper), and (*b*) dams and *Macrobrachium* spp. world distributions.

We estimated that 277–385 million people at risk of schistosomiasis also live within the native range of one or more large, marketable *Macrobrachium* spp. prawns. This represents 33–46% of the global population at risk of schistosomiasis. The native range of a single West African prawn species *M. vollenhovenii*—alone—contains 136–186 million people at risk of schistosomiasis (31–42% of the total sub-Saharan African at-risk population). Some areas in central Africa and southern Brazil are schistosomiasis-endemic but do not overlap with any migratory river prawn habitats (figure 3). In addition, no large native *Macrobrachium* spp. river prawn habitats are reported from schistosomiasis-endemic areas in North Africa or the Middle East. However, despite these prominent geographical gaps in prawn–schistosomiasis overlap, one-third to one-half of the global population at risk of schistosomiasis was estimated to live in historical prawn habitat, perhaps due to the demographic affinity of human settlements for coastal communities, where prawn habitats are common.

**Figure 4.**
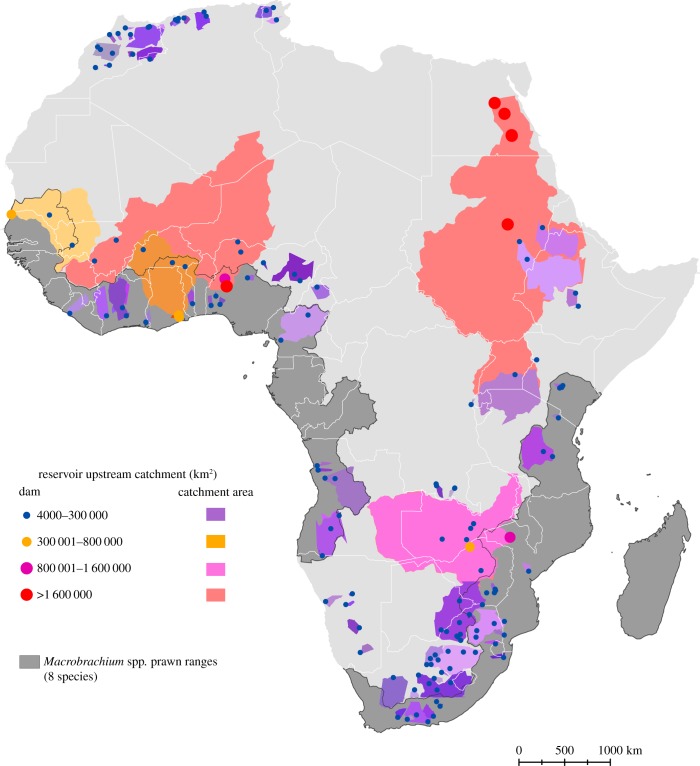
Map showing locations of dams (coloured circles), upstream dam catchments (coloured regions), and *Macrobrachium* spp. prawn distributions (dark grey regions) on the African continent. Dam data were sourced from the freely available GRaND database: http://www.gwsp.org/products/grand-database.html.

More than 33 000 dams are listed in the World Register of Dams [42], with 6862 of the largest dams georeferenced in the Global Reservoir and Dam (GRanD) Database [38] (figure 3*b*). Visualizing the upstream catchments of large dams on the African continent (figure 4), shows many dam catchments overlapping with, and presumably blocking access to, much of the river networks once inhabited by migratory prawns. Parts of West Africa, East Africa and southeastern Africa harbour the highest densities of large dam catchments overlapping historical prawn habitats. These areas also contain very high schistosomiasis prevalences.

## 4. Discussion

Our results support the long-recognized association between schistosomiasis and dams, and indicate that, across many of the 14 dams evaluated, the increase in schistosomiasis after dam-building was dramatic. We further show that the effects of dams on schistosomiasis extend far beyond the reservoirs created above dams, areas which have been the focus of previous large studies. In fact, upstream catchments hundreds of kilometres from dams often experienced schistosomiasis increases after dam construction to nearly the same degree as did the populations near the reservoirs immediately upstream of the dams (figure 2).

Schistosomiasis increased more within prawn ranges (OR 2.8 and 4.4 for *S. haematobium* and *S. mansoni*, respectively) than outside prawn ranges (OR 1.15 and 1.5 for *S. haematobium* and *S. mansoni*, respectively), and schistosomiasis decreased overall in the undammed, control watersheds (OR 0.9 and 0.75 for *S. haematobium* and *S. mansoni*, respectively). In other words, dam effects on schistosomiasis were stronger when catchments lost their migratory snail predators (such as river prawns). This association suggests that the presence of migratory prawn predators of snails may limit transmission of schistosomiasis.

Although we observed that the probable loss of prawns was associated with increases in human schistosomiasis, the prawn effect is confounded with other ecological and socio-economic effects. In the Senegal River Basin, post-dam increases in schistosomiasis have been anecdotally attributed to a reduction in flow and salinity and a concomitant increase in pH in the lower Senegal River Basin [2,43]. Hypothetically, lowered salinity, due to blocked tidal influence upstream during the dry season, might have increased the availability of freshwater habitat for snails (because even low salinity levels are detrimental to *Biomphalaria* and *Bulinus* spp. survival and reproduction [44]). More freshwater habitat, in turn, promoted aquatic plant and algal growth [45], thereby increasing food availability and refuge for snails. However, the extent of the pre-dam tidal influence in the Senegal River Basin penetrated to only approximately 120 km inland [46], whereas the *S. haematobium* increases after dam construction extended well beyond this limit, to habitats that were suitable for snails before and after the dam. Thus, salinity change does not provide a general explanation for the widespread schistosomiasis increases observed both near and far from this coastal dam. Furthermore, another coastal dam within prawn range, Akosombo, in Ghana, probably did not cause salinity changes upstream, because tidal influence in this basin only extends to 40 km inland [47], and the Akosombo Dam sits 105 km inland. Salinity shifts above this dam are therefore an implausible explanation for the observed schistosomiasis increases. This begs the question: what other dam-associated ecological changes occurred here and across the other coastal dams that might provide an alternative explanation for schistosomiasis increases?

The fact that schistosomiasis decreased in undammed (coastal) watersheds makes it unlikely that unmeasured factors (unrelated to dams) caused schistosomiasis to systematically increase more on the coast than inland. Other possible dam effects – not related to a loss of migratory predators – probably occurred above dams both inside and outside prawn ranges, such as increases in aquatic vegetation, inundation, agricultural inputs, worker migrations, or water contact. Whether these or other dam-associated ecological changes were systematically different above the more coastal dams within prawn ranges compared to the more inland dams outside prawn ranges warrants further exploration.

Our study begins to address the lack of historical emphasis on the ecological mechanisms underlying dam-driven schistosomiasis outbreaks, which has led to ongoing failures to prevent or mitigate the health risks of water resources development [13]. Our results support the idea that prawn restoration is a generalizable, environmentally sound option for schistosomiasis control wherever river prawns are native and people at risk of schistosomiasis live in watersheds that overlap with prawn habitat. We estimate that one third to one half of the global population at risk of the disease, or 277–385 million people live in schistosome-endemic watersheds that also overlap with prawn habitat (figure 3*a*).

Our geographical correlations are consistent with laboratory, field and modelling studies demonstrating that prawns can control snails effectively and that their restoration can improve the efficacy of mass drug administration-based schistosomiasis control programmes [5,7]. Prawn restoration would be most effective if deployed in combination with traditional medical interventions that notably reduce morbidity and anthelmintics that treat existing worm burdens.

The effectiveness and scalability of restoring prawns to reduce schistosomiasis remains incompletely assessed. This would require a set of large-scale trial interventions. Our analysis reveals some promising candidate regions for such interventions in sub-Saharan Africa, including the Senegal River Basin, the Volta River Basin, the Masinga and Kiambere catchments in Kenya, the Mbakaou and Taabo catchments in Cote D'Ivoire, the Nangbeto catchment in Togo, and the Oyan catchment in Nigeria. There are probably many other areas of Africa, and possibly the Americas or Asia, where opportunities exist to test prawn restoration as an ecological approach to disease control. Dams are not the only places where the nexus of increased habitat for snails and snail-predator exclusion can lead to increased schistosomiasis. For example, the widespread development of man-made irrigation schemes that both increase inundation and support snail habitat, while excluding large aquatic snail predators, could also create an unfortunate opportunity for intensified schistosomiasis transmission.

The most significant challenge to controlling schistosomiasis with prawns rests on bringing governments and engineers into interdisciplinary collaborations with health specialists and ecologists. Such collaborations will be crucial in efforts to build new dams or retrofit old ones, to ensure that aquatic animal migrations are maintained, biodiversity is preserved, and human health is safeguarded.

There are two obvious ways to restore river prawns while maintaining dams. First, juvenile prawns can scale waterfalls, suggesting that prawn passages could be engineered into dams in the same way that eel ladders have helped to restore upstream eel populations [15,48]. Second, prawn stocking through aquaculture might be an effective tool in managed ecosystems such as irrigation canals. Prawn aquaculture is a lucrative business in its own right [49]. Add the benefit of reduced schistosomiasis wherever prawns are stocked, and cultivating prawns in these agricultural schemes could be beneficial for both agricultural workers and the wider communities residing near such managed landscapes. In Asia, polyculture of prawns in rice schemes is already a common agricultural activity [50,51]. If this could be extended to the agricultural landscapes of Africa and the Americas where schistosomiasis transmission is highest, prawn aquaculture might offer a powerful tool in the global fight against schistosomiasis. The schistosomiasis case study demonstrates how targeted ecosystem restoration (prawn reintroduction) could benefit both human health and biodiversity. Ecological, health, and socio-economic effects of development projects like dams can have complex feedbacks, such as the circular effects of wealth (and urbanization) on reducing infectious disease risk [52] and of infectious disease risk on wealth and the perpetuation of poverty [53]. Where these feedbacks exist, interdisciplinary interventions that consider both human health and environmental effects could be beneficial. A future question remains: for what other human disease systems can win–win solutions for people and nature be carried out successfully at scale, while remaining cost-effective? For example, a similar strategy of predator (large carnivore) restoration in terrestrial habitats of North America has been suggested as a potential tool to reduce rodent density and therefore Lyme disease risk for people [54]. There may be untapped opportunities to devise health-oriented ecological solutions to human health problems wherever human pathogens, such as schistosomiasis, pass obligately through the natural environment in order to be transmitted [53].

## Supplementary Material

Supplemental Figure 1

## Supplementary Material

Supplemental Figure 2
