# Supplementary material for: Nearly 400 million people are at higher risk of schistosomiasis because dams block the migration of snail-eating river prawns
Source: Philos Trans R Soc Lond B Biol Sci. 2017 Apr 24;372(1722):20160127. doi: 10.1098/rstb.2016.0127 (PMC5413875; doi:10.1098/rstb.2016.0127)

Supplemental Figure 1. Modeled prawn habitat ranges and Global Biodiversity Information Facility's (GBIF) georeferenced sightings for the *Macrobrachium* prawn species assessed in this study. The proposed 400km and 1000km ranges were strongly associated with *Macrobrachium* sightings from the GBIF database ($p<0.001$), with 81% of sightings reported within the 400km range and an additional 10% of sightings between the 400km and 1000km ranges. The estimated intensity of sightings per unit area between 400km and 1000km from the coast was 6.4 times (95% CI: 4.0 – 10.3) higher than in areas located further inland, while the intensity of sightings within the 400km range was 30.4 times (95% CI: 21.3 – 43.3) higher than the intensity in areas outside of the proposed ranges. The sighting rate in the 400km range was also significantly higher than that in the 1000km range ($p<0.001$), suggesting a decreasing gradient of prawn abundance moving further inland. We determined that *M. jeslkii*, a South American species with abbreviated larval development, is capable of breeding in freshwater. It is likely that some *M. jelskii* sightings beyond our predicted ranges were non-migratory, freshwater adapted populations, and therefore we conducted analyses both including and excluding *M. jelskii* sightings (red arrows). When *M. jelskii* was excluded from analyses, 83% of all sightings fell within the 400km range and intensity was 37.9 times (95% CI: 31.0 – 46.3) greater than outside of either range, while areas between the 400km and 1000km ranges encompassed an additional 10% of sightings, with intensity 7.9 times (95% CI: 6.0 – 10.2) greater.

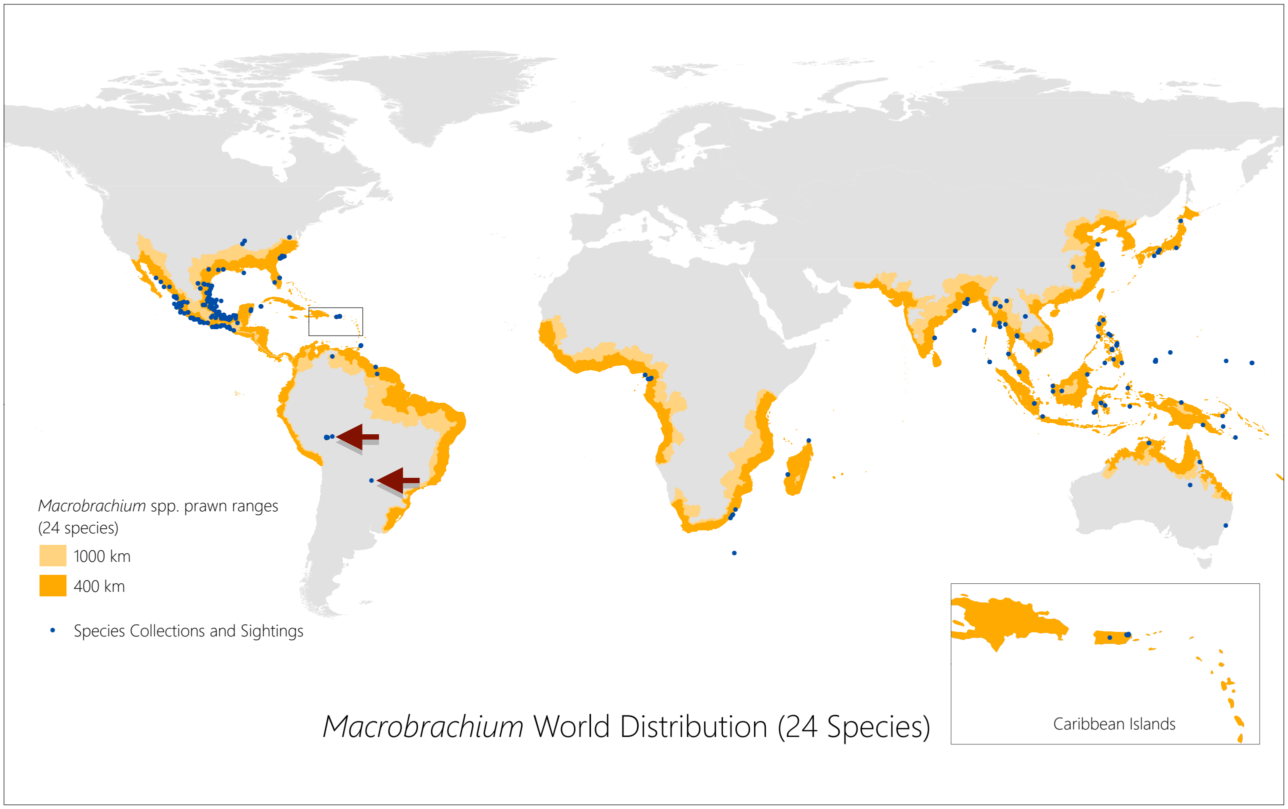

Supplement: Supplemental Figure 1 [file rstb20160127supp1.pdf]
