# Supplementary material for: Nearly 400 million people are at higher risk of schistosomiasis because dams block the migration of snail-eating river prawns
Source: Philos Trans R Soc Lond B Biol Sci. 2017 Apr 24;372(1722):20160127. doi: 10.1098/rstb.2016.0127 (PMC5413875; doi:10.1098/rstb.2016.0127)

Supplemental Figure 2. Global map of estimated river prawn species richness for the 24 migratory *Macrobrachium* spp. assessed in this study.

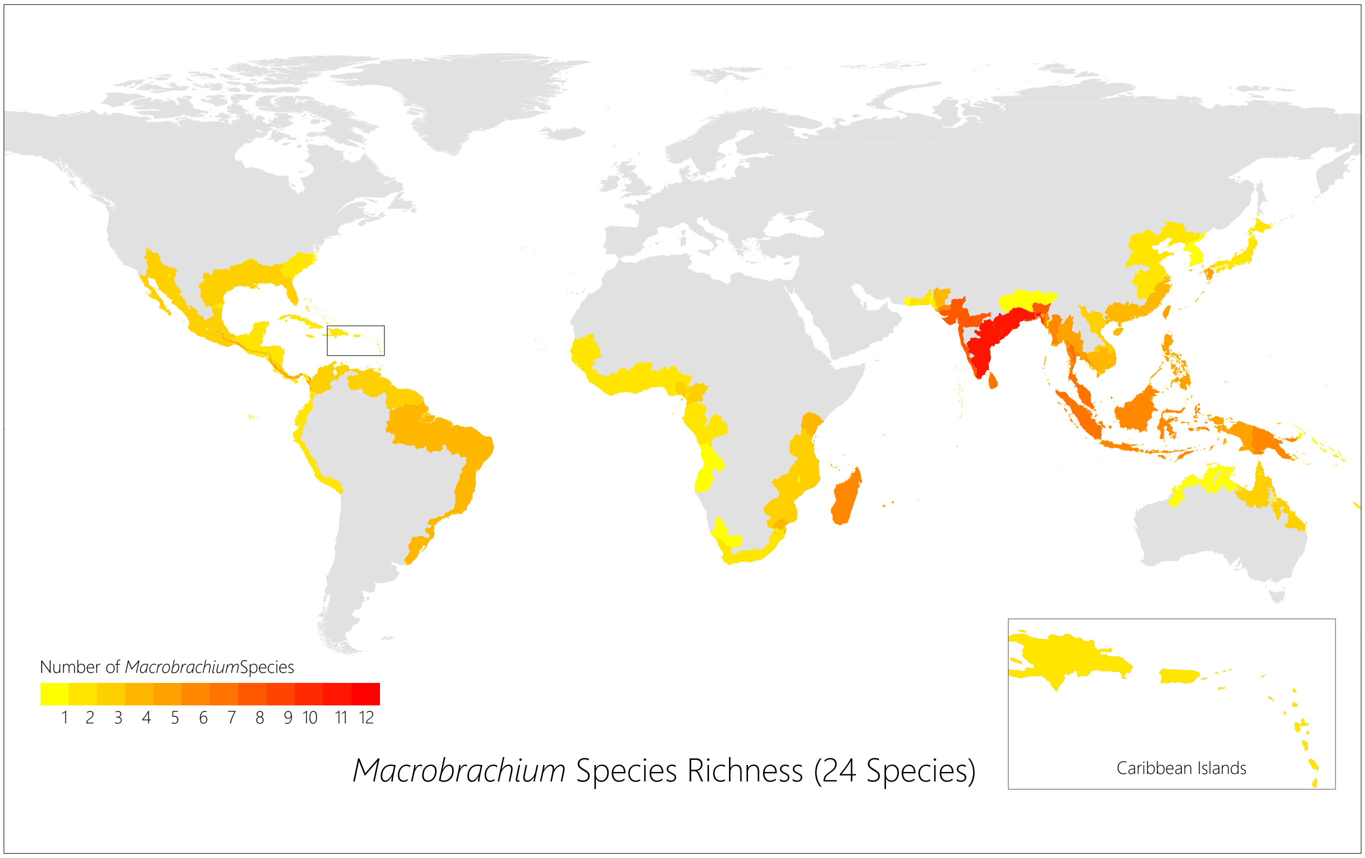

Supplement: Supplemental Figure 2 [file rstb20160127supp2.pdf]
